# Multicenter DSC–MRI-Based Radiomics Predict IDH Mutation in Gliomas

**DOI:** 10.3390/cancers13163965

**Published:** 2021-08-05

**Authors:** Georgios C. Manikis, Georgios S. Ioannidis, Loizos Siakallis, Katerina Nikiforaki, Michael Iv, Diana Vozlic, Katarina Surlan-Popovic, Max Wintermark, Sotirios Bisdas, Kostas Marias

**Affiliations:** 1 Computational BioMedicine Laboratory (CBML), Foundation for Research and Technology—Hellas (FORTH), 70013 Heraklion, Greece; geo3721@ics.forth.gr (G.S.I.); kat@ics.forth.gr (K.N.); kmarias@ics.forth.gr (K.M.); 2 Department of Brain Repair and Rehabilitation, Queen Square Institute of Neurology, UCL, London WC1N 3BG, UK; loizos.siakallis.20@ucl.ac.uk (L.S.); s.bisdas@ucl.ac.uk (S.B.); 3 Division of Neuroimaging and Neurointervention, Department of Radiology, Stanford University, Stanford, CA 94305, USA; miv@stanford.edu (M.I.); mwinterm@stanford.edu (M.W.); 4 Department of Radiology, Faculty of Medicine, University of Ljubljana, 1000 Ljubljana, Slovenia; diana.vozlic@kclj.si (D.V.); katarina.surlan@kclj.si (K.S.-P.); 5 Department of Neuroradiology, University Medical Centre, 1000 Ljubljana, Slovenia; 6 Department of Neuroradiology, The National Hospital for Neurology and Neurosurgery, University College London NHS Foundation Trust, London WC1N 3BG, UK; 7 Department of Electrical & Computer Engineering, Hellenic Mediterranean University, 71410 Heraklion, Greece

**Keywords:** dynamic susceptibility contrast MRI, gliomas, radiomics, IDH mutation, generalizability, explainability, external validation

## Abstract

**Simple Summary:**

Significant efforts have been put toward developing MRI-based radiogenomics for IDH status subtyping predictions; however, in the vast majority of these approaches, the external validation sets are absent. Another limitation in current studies is the lack of explainability in radiomics models, which hampers clinical trust and translation. Motivated by these challenges, we proposed a multicenter DSC–MRI-based radiomics study based on an independent exploratory set, which was externally validated on two independent cohorts, for IDH mutation status prediction. Our results demonstrated that DSC–MRI radiogenomics in gliomas, coupled with dynamic-based image standardization techniques, hold the potential to provide (a) increased predictive performance by offering models that generalize well, (b) reasoning behind the IDH mutation status predictions, and (c) interpretability of the radiomics features’ impacts in model performance.

**Abstract:**

To address the current lack of dynamic susceptibility contrast magnetic resonance imaging (DSC–MRI)-based radiomics to predict isocitrate dehydrogenase (IDH) mutations in gliomas, we present a multicenter study that featured an independent exploratory set for radiomics model development and external validation using two independent cohorts. The maximum performance of the IDH mutation status prediction on the validation set had an accuracy of 0.544 (Cohen’s kappa: 0.145, F1-score: 0.415, area under the curve-AUC: 0.639, sensitivity: 0.733, specificity: 0.491), which significantly improved to an accuracy of 0.706 (Cohen’s kappa: 0.282, F1-score: 0.474, AUC: 0.667, sensitivity: 0.6, specificity: 0.736) when dynamic-based standardization of the images was performed prior to the radiomics. Model explainability using local interpretable model-agnostic explanations (LIME) and Shapley additive explanations (SHAP) revealed potential intuitive correlations between the IDH–wildtype increased heterogeneity and the texture complexity. These results strengthened our hypothesis that DSC–MRI radiogenomics in gliomas hold the potential to provide increased predictive performance from models that generalize well and provide understandable patterns between IDH mutation status and the extracted features toward enabling the clinical translation of radiogenomics in neuro-oncology.

## 1. Introduction

Gliomas consist of a heterogeneous group of tumors and account for the majority of malignant primary brain tumors [1]. Histological grading and classification into low- and high-grade gliomas [2] has been limited in deciphering the heterogeneous treatment response and survival of glioma patients [3], apart from predicting worse clinical outcomes in patients with glioblastoma [4]. Molecular and genetic analyses yielded distinct biochemical and genetic traits of glioma subgroups beyond histological grading [3]. Of these, isocitrate dehydrogenase (IDH) is probably the most important in terms of prognostication [5]. The identification of an IDH mutation in low- and high-grade gliomas and its role in gliomagenesis elucidate the progression of low-grade glioma (LGG) to an IDH-mutant, “secondary” glioblastoma via a common pathway [6]. While IDH-mutant tumors are favored for their improved clinical outcomes, their IDH-mutant counterparts are characterized by a dismal prognosis, irrespective of histological grading [7]. Due to its propensity to stratify glioma patients, IDH mutations were identified in the recent world health organization (WHO) classification as a distinctive feature of glioma subgroups [3].

Characterization of the IDH mutation status has therefore become imperative prior to treatment selection and patient stratification [8]. However, this still relies on invasive tissue sampling and analysis, harboring inherent limitations, including post-operative complications and sample error [9]. The non-invasive classification of gliomas based on imaging was proposed as an alternative to this approach, leading to the identification of imaging features as correlates to molecular subtypes. Structural features, including calcification and T2-FLAIR mismatch and tumor enhancement, yielded promising, though inconsistent, outcomes for the identification of IDH status [10]. This was partly attributed to interobserver variability during the visual assessment of such features [11]. To this end, machine learning (ML) enhanced the identification of high-throughput quantitative imaging features that were extracted from conventional and multiparametric magnetic resonance imaging (MRI) was successfully applied for the characterization of IDH mutation status, with promising outcomes outlined in recent radiogenomics studies [12]. Results that were found using contrast-enhanced T1-weighted, T2-weighted, and arterial spin labeling (ASL) images from 105 glioma patients showed an accuracy of 0.82 and an area under the curve (AUC) of 0.77 on internal validation using an iterated cross-validation [13]. A recent review of radiomics implications in glioma [14] produced an accuracy of 0.83 when multicenter multiparametric MRI samples were divided randomly into training and validation sets [15], and an accuracy of 0.99 was found when T2-weighted MRI-based radiomics were applied to training and validation sets from a single center [16]. An accuracy of 0.917 (sensitivity: 0.857, specificity: 1) was achieved in [17] using a three-level radiomics model based on multiparametric MRI (postcontrast T1-weighted, T2-weighted, T2-FLAIR, and diffusion-weighted imaging) obtained from the Cancer Imaging Archive (TCIA) and validated on a cohort of 12 patients. Finally, a random forest showed an accuracy of 0.885 ± 0.041 and an AUC equal to 0.931 ± 0.036 when tested on multiparametric MRI data using an iterated training/testing split from 126 patients [18]. Although several studies, as presented in a recent review paper [12] managed to assess the value of conventional and multiparametric MRI in predicting glioma IDH mutation status, a lack of independent sets for validating externally related radiomics signatures was evident in most of the studies, as denoted by the radiomics quality score (RQS) [19]. This limitation also concerns neuro-oncology radiomics in general, where only 3.9% (2/51) of studies were found to be validated using multicenter data [20].

Potential imaging biomarkers beyond structural features were highlighted by the long recognized tumor vascularity’s correlation with histological grade [21], and the role of dynamic susceptibility contrast (DSC) perfusion was noted by the recent identification of distinct angiogenesis transcriptome signatures of IDH-mutant gliomas linked to identifiable perfusion phenotypes [22]. However, not much research has investigated the integrative role of DSC perfusion, as opposed to standalone structural MRI, in radiogenomics toward the non-invasive characterization of IDH status based on distinct vascular profiles [22]. More specifically, to the best of our knowledge, IDH status mutation prediction is limited to a single study from [23] in which repeated cross-validations, performed on a unique cohort of six tertiary centers, reported an accuracy of 0.72 (overall specificity: 0.77, sensitivity: 0.65). This was further confounded by variations in DSC perfusion acquisition and analysis, despite current attempts for consensus guidelines [24]. Another limitation in current studies is the lack of explainability in radiomics models, which hampers clinical trust and translation to neuro-oncology clinical practice.

In an effort to address all the concerns stated above, this study aimed to explore the potential of DSC–MRI-based radiomics to predict IDH mutation status in gliomas. The proposed radiogenomics analysis was built using an independent exploratory set, a dynamic-based standardization of the MR images, and an external validation from two independent cohorts. To interpret the IDH mutation status predictions, model explainability was performed using local interpretable model-agnostic explanations (LIME) and Shapley additive explanations (SHAP). To the best of our knowledge, this is the first attempt toward validating a DSC–MRI radiogenomics study on independent external cohorts and interpreting the corresponding IDH mutation status prediction results for gliomas.

## 2. Materials and Methods

### 2.1. Patient Population

The studied population included 160 patients (age: 58.4 ± 15.9 (mean ± SD), 70 female) from 3 tertiary centers, each with a histopathological diagnosis of primary glioma (WHO grades 2–4), molecular characterization of IDH mutation status (IDH-mutant = 41, IDH-wildtype = 119) and DSC–MRI data prior to any treatment. Specifically, cohort A consisted of 92 patients (66 out of 92 IDH-mutant), cohort B included 50 patients (39 out of 50 IDH-mutant), and cohort C contained 14 out of 18 patients with an IDH-mutant status. We excluded patients without a histologically confirmed diagnosis of glioma, incomplete molecular characterization of IDH status, non-enhancing anaplastic grade III gliomas, or patients having received any treatment prior to image acquisition. The study was approved by each local ethics committee and all patients signed informed consent forms prior to examination.

### 2.2. MRI Protocol

Patients underwent DSC–MRI from different scanners and acquisition protocols. The imaging parameters for each cohort are presented in Table 1.

### 2.3. Tumor Delineation

Lesion voxels of interest (VOIs) were automatically generated using Bratumia [25] software (https://www.nitrc.org/projects/bratumia, accessed on 24 June 2021), which uses four different contrasts (T2, T2 FLAIR, T1 before and after contrast) as input in order to correctly identify tumor enhancement, edema, and necrosis from normal brain tissue. Two expert neuroradiologists with 7 and 4 years of experience, respectively, visually inspected the generated tumor voxels of interest (VOIs) and provided corrections when necessary on a consensus basis. 

### 2.4. Time Point Selection

**Figure 1:**
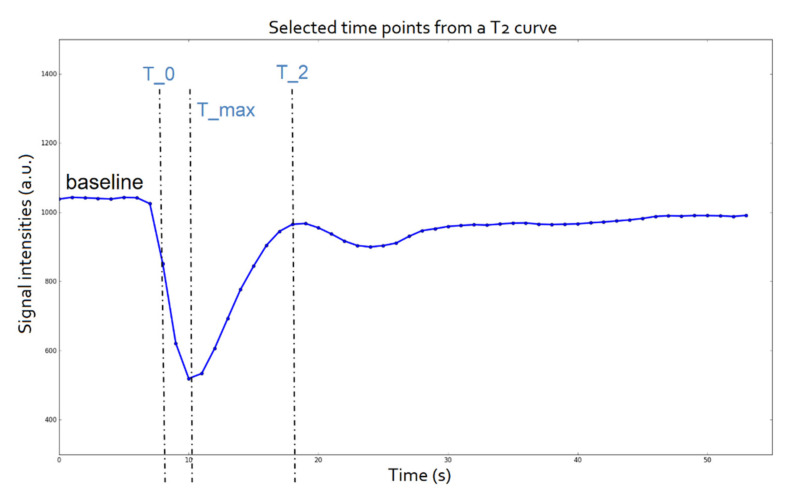
Time points of interest of a T2 mean signal intensity curve.

After the tumor delineation, the mean signal intensity from all voxels assigned as a lesion were used to create the signal intensity curve of each patient over time. DSC–MRI data were inspected for an adequate number of time points to establish the baseline (minimum 5) before the maximum signal drop resulting from the arrival of the contrast agent to the voxels in question. Three characteristic points of interest (T_0, T_max, and T_2), defined as the time of the first measurement after contrast detection, time of maximum signal drop, and time of return to baseline, respectively, were selected for each specific patient from their corresponding signal intensity curve over time (Figure 1).

### 2.5. Dynamic-Based Standardization of the MRI Data

**Figure 2:**
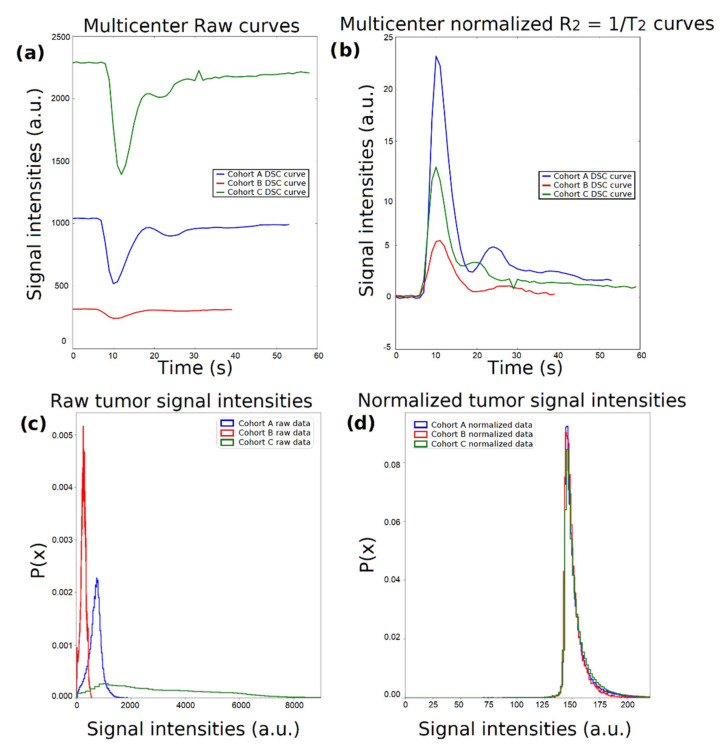
Schematic representation of the normalization process. (**a**) Dynamic susceptibility contrast (DSC) perfusion curves from the 3 tertiary centers. (**b**) $\Delta R_{2}\left(t\right)$ curves after normalization using equation (1). (**c**) Center-specific probability density functions of the raw signal intensities within the voxels of interest (VOIs) at time point T_max. (**d**) Center-specific probability density functions of the normalized signal intensities within the VOIs at time point T_max.

Considering that the MR signal is measured in arbitrary units, it is challenging to perform joint analyses from data originating from different clinical centers since the image intensities exhibit inherent variability and different ranges of values. Indicative perfusion curves from each patient cohort are presented in Figure 2a, where the differences in the range of the exported MR image intensity values are evident. Supposing a DSC perfusion curve over time $T_{2}\left(t\right)$ or the relaxation rate curve $R_{2}\left(t\right)=1/T_{2}\left(t\right)$, the first step was the baseline correction, which was achieved by subtracting the last signal of the baseline ($T_{2}\left(0\right)$, contrast agent enters the vasculature system) from each curve as follows: $\Delta T_{2}\left(t\right)=T_{2}\left(t\right)\;-T_{2}\left(0\right)$ (Figure 2b). As a second step, due to the variability between different protocols with respect to echo time (*TE*), each curve was modified to account for the exponential signal decay in time by using Equation (1):

(1) $$\Delta R_{2}\left(t\right)=\frac{1}{TE}ln\frac{{\bar{T_{2}\left(t\right)}}_{baseline}}{\Delta T_{2}\left(t\right)}\left(1\right)$$

where ${\bar{T_{2}\left(t\right)}}_{baseline}$ is the mean baseline of the raw signal $T_{2}\left(t\right)$ [26]. Moreover, signal intensity probability density functions for all voxels annotated as a tumor per imaging group of patients for the maximum enhancement time point are presented for the raw (Figure 2c) and the normalized (Figure 2d) $T_{2}\left(t\right)$ data.

### 2.6. Radiomics Analysis Workflow

**Figure 3:**
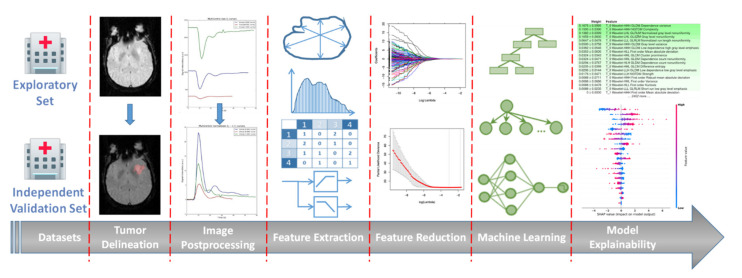
An illustrative representation of the proposed radiomics analysis workflow.

The proposed analysis workflow is illustrated in Figure 3 and presented in detail below.

#### 2.6.1. Image Postprocessing

**Figure 4:**
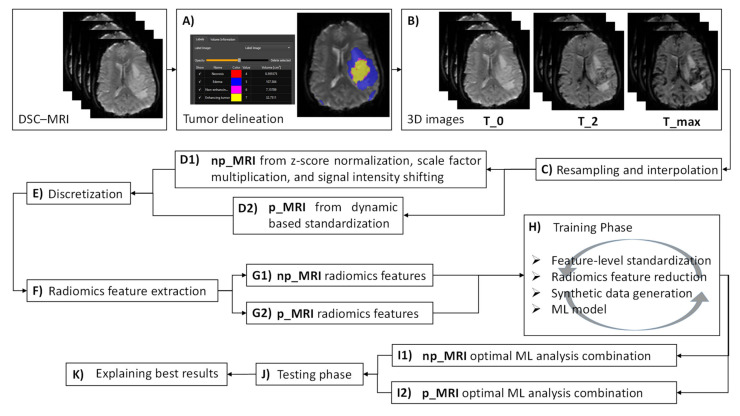
A schematic diagram summarizing the proposed radiomics analysis workflow steps.

Initially, tumor delineations were automatically generated as reported in Section 2.3 (step A, Figure 4) and corresponding 3D images at the specific time points T_0, T_max, and T_2 were extracted from the dynamic acquisition data (Section 2.4 and step B from Figure 4). Subsequently, 3D images were spatially resampled and interpolated (step C, Figure 4) to compensate for multicenter effects (e.g., differences in the acquisition protocol and the reconstruction settings), enabling textural feature extraction in the 3D domain since rotation-invariant 3D images are recommended. Although there is no clear suggestion of whether to use a downsampling or upsampling interpolation, volumes in this study were resampled to isotropic voxels of length 1 mm using the cubic B-spline interpolation method.

To assess the potential impact of the proposed standardization technique (Section 2.5) on the IDH status prediction, two different versions of the extracted 3D images were generated. In the first version (step D1, Figure 4), the signal intensities from the 3D images (hereafter denoted as np_MRI) were z-score normalized using the mean and standard deviation from all voxels within the VOIs drawn on the overall DSC–MRI data (VOIs drawn across all time points to keep the dynamic changes of the tissue over time). Next, normalized signals from np_MRI data were multiplied by a scaling factor that was set to 100 and shifted by the minimum signal intensity that was derived from np_MRI histograms to ensure that all voxels within the VOIs had positive values. In the second version (step D2, Figure 4), the 3D images (hereafter denoted as p_MRI) were dynamically standardized (Section 2.5). At the next step, both np_MRI and p_MRI data underwent discretization (step E, Figure 4) to account for differences in the voxel intensity ranges using Equation (2), where *FBS* is the fixed bin size, *FBN* is the fixed bin number, and *mean_Rang_*_e_ is the derived mean intensity range from the VOIs histogram of all patients within the examined cohort. In the current analysis, *FBN* was set to 32 and 64 since a low number of bins is recommended in brain MRI radiomics [27].

(2) $$FBS=\frac{1}{FBN}\times mean_{Range}$$

#### 2.6.2. Radiomics Feature Extraction

Radiomics feature extraction (step F, Figure 4) was carried out in compliance with the image biomarker standardization initiative (IBSI) guidelines. A total of 833 features were automatically extracted from the segmented regions of every image (both np_MRI and p_MRI) using pyradiomics [28] comprising features: (a) without any filter applied before the extraction and (b) after images were decomposed using a wavelet transform (Coiflet 1) of level 1. Among the non-wavelet features (105 features, summarized in Supplementary Table S1): 16 first-order statistics were calculated from the histogram of the VOIs (group 1); 14 morphological features contained information about the shape and size of the lesion (group 2); 24 s-order statistics (group 3) computed from the grey level co-occurrence matrix (GLCM); and 14, 16, 16, and 5 third-order statistics on the gray level dependence matrix (GLDM), gray level size zone matrix (GLSZM), gray level run length matrix (GLRLM), and neighboring gray tone difference matrix (NGTDM), respectively (group 4). Additionally, to address the multi-scale nature of texture, 728 features from groups 1, 3, and 4 were derived using a set of eight sub-bands of the decomposed images (HHH, HHL, HLH, HLL, LHH, LHL, LLH, and LLL) with different resolutions corresponding to the low- (L) and high-pass (H) filters applied along the X-, Y, and Z-axis (91 features × 8 sub-bands). In total, 2499 (833 × 3, where three corresponds to the three time points) np_MRI (step G1, Figure 4) and 2499 p_MRI related radiomics (step G2, Figure 4) features were integrated into a single feature vector, respectively.

#### 2.6.3. Machine Learning Analysis Framework 

A machine learning analysis pipeline was designed comprising: (a) feature-level standardization, (b) radiomics feature reduction, (c) synthetic data generation, and (d) the application of the ML model. Concerning (a), a univariate analysis was initially conducted using the Shapiro–Wilk test to assess the normality of the radiomics features. Most of the features achieved a *p*-value less than 5%, indicating a non-normal distribution. Therefore, centering and scaling of all exported radiomics features was performed with “RobustScaler” [29] since it is less prone to outliers and can operate on non-normal distributed data. Several feature reduction techniques performed during (b) were utilized to decrease the high-dimensional radiomics feature vector by considering their low computational complexity and their effectiveness, as reported in similar radiomics studies. These included univariate filtering techniques (Gini Index, F-ratio, information gain, and Spearman correlation) and a multivariate feature scoring criterion using: (a) the minimum-redundancy maximum-relevance (mRMR), (b) mutual information maximization (MIM), and (c) the statistical inference relief (STIR); all of these were tested with a pre-selected number of features varying from 5 to 50. Additionally, the least absolute shrinkage and selection operator (LASSO) regression was also examined to identify a subset of informative radiomics features (features with nonzero coefficients) using the L1 norm penalty and a logistic regression with elastic net regularization, combining the LASSO and ridge regression with a ratio of 0.5 between the L1 and L2 penalties.

Considering the highly unbalanced ratio between the two examined classes, different synthetic data generation techniques based on data oversampling, undersampling, and a combination of over- and undersampling were applied during the model training to manage the class unbalancing. To this end, five different approaches were examined: synthetic minority oversampling technique (SMOTE), adaptive synthetic (ADASYN), SMOTE followed by cleaning using Tomek links (SMOTE-Tomek), random undersampling (RUS), and undersampling using the Nearmiss method (NearMiss). Additionally, a no-resampling choice was added as an option to the analysis pipeline. For the application of the ML model, the current study utilized a support vector machine (SVM) with a linear kernel; a random forest (RF); a K-nearest neighbor (KNN); a logistic regression with L2, elastic net, and L1 norm penalties; and adaptive boosting (AdaBoost) using a decision tree as the base classifier. Additional ensemble learning techniques were initiated, comprising a RUSBoost (an AdaBoost coupled with a random undersampling) with a decision tree as the base classifier, a gradient boost (XGBoost), and bagged classification trees (BAG) integrated with resampling of the majority class (IDH-mutant). When RUSBoost and BAG were operated, no resampling was applied. Feature-level optimization was implemented using scikit-learn [29]; radiomics feature reduction was developed using scikit-learn [29] and ITMO FS (https://github.com/ctlab/ITMO_FS, accessed on 24 June 2021); imbalanced-learn [30] was utilized for synthetic data generation; and packages xgboost (https://github.com/dmlc/xgboost, accessed on 24 June 2021), scikit-learn [29], and imbalanced-learn [30] contributed in the development of the ML models.

#### 2.6.4. Performance Validation and Explainability of the Predictions

The ML analysis framework (Section 2.6.3) was initially applied to both the np_MRI and p_MRI data from cohort A (exploratory set) and subsequently validated externally using np_MRI and p_MRI data from cohorts B and C (validation set). An internal validation according to a stratified five-fold repeated cross-validation with two hundred repeats was conducted during the training phase using the exploratory set (step H, Figure 4). Identical results regarding the samples of each fold were subsequently used from every possible combination of the analysis steps in both cases (np_MRI and p_MRI). An average estimate of Cohen’s kappa metric was then obtained at the validation phase (within the internal validation) as a criterion to determine the highest performance among all combinations. Cohen’s kappa was chosen as the preferable performance metric to select the optimal ML analysis combination since it considers class unbalancing in the prediction accuracy. To eliminate any bias during training, the feature-level standardization, radiomics feature reduction, and synthetic data generation from Section 2.6.3 were applied exclusively at the training phase. In all cases, the ML models defined in Section 2.6.3 were initialized using the default parameters. For both the np_MRI and p_MRI data, the combination that showed the highest performance with respect to the Cohen’s kappa (steps I1 and I2 from Figure 4) was then tested in the independent test set (step J, Figure 4). Model performance was also assessed using the F1-score, accuracy, AUC, sensitivity, specificity, positive and negative predictive value (PPV and NPV). The IDH-wildtype was considered as the positive class. Apart from constructing generalizable ML models, the explainability and interpretability of their decisions are the key milestones of increased impact toward improving the clinical translation of radiogenomics studies. To this end, the present study developed a postprocessing phase using SHAP [31] and LIME [32] methodologies to provide reasoning behind the IDH mutation status predictions and interpret the impact of each radiomics feature in the model’s performance (step K, Figure 4). According to LIME, once a model is trained on the exploratory set, feature permutation importance (reported as mean ± SD) is then computed on the validation set by shuffling all corresponding features individually. In our analysis, this yielded a top-down radiomics feature order in decreasing importance, which was related to the reshuffling of each particular feature from the ordered list. To further illustrate the interpretability of the radiomics analysis pipeline that showed the highest predictive performance regarding the IDH status, a SHAP explainer was designed on the exploratory set and the distribution of features’ impact on the predictions was then calculated based on the validation set and displayed using the SHAP summary plot.

## 3. Results

**Figure 5:**
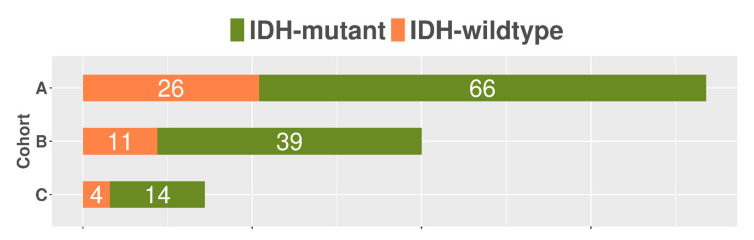
Distribution of the isocitrate dehydrogenase (IDH) status across the three examined centers. Numbers of total IDH-mutant and IDH-wildtype statuses from each clinical center are shown in white.

A multicenter radiogenomics study was conducted using imaging data that was acquired from different vendors, who used different magnetic strengths and acquisition protocols. Overall, cohort A included 60 males and 32 females with a mean and standard deviation (SD) age of 57 (15.1) years, cohort B was composed of 28 males and 22 females (age in years: mean = 58, SD = 17.1), and 9 males and 9 females belonged to cohort C with a mean age of 55.7 years and a standard deviation equal to 15 years. No statistically significant changes were observed between the ages of the three investigated cohorts. A high-class imbalance of the IDH status of the patients was evident in all examined cohorts, as depicted in Figure 5.

Several combinations of all techniques reported in Section 2.6.3 were developed using a total of 2499 radiomics features from cohort A and validated on cohorts B and C using Cohen’s kappa metric. Combinations having a mean Cohen’s kappa equal to or lower than the baseline (i.e., a zero value in the case of random model’s accuracy) were discarded from the validation phase. In the case of the radiomics analysis on the np_MRI, the best model training performance was obtained when the features were:

1. Normalized with “RobustScaler”;
2. Reduced to the 15 most significant features using univariate filtering according to the Gini Index;
3. Subjected to synthetic minority oversampling based on the ADASYN;
4. Providing input to an elastic net logistic regression model, yielding a mean Cohen’s kappa of 0.193 (SD: 0.096) using the exploratory set and 0.145 when validated externally.

**Figure 6:**
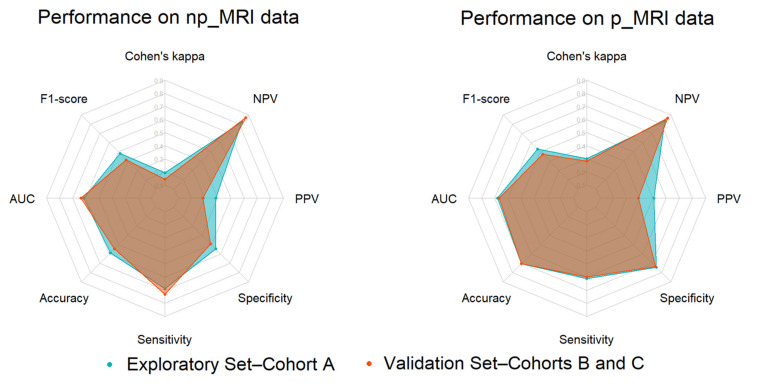
From left to right: Radar plots illustrating the IDH mutation status prediction performance during the model development (internal validation using the exploratory set) and validation (external validation set) phases from np_MRI and p_MRI data.

The p_MRI radiomics analysis performance was found to be significantly higher, yielding a Cohen’s kappa for the exploratory and the validation set equal to 0.303 ± 0.125 (mean ± SD) and 0.282, respectively. Among all possible combinations, the optimal p_MRI radiomics analysis pipeline was constructed from “RobustScaler”, which is a multivariate feature scoring criterion that is based on MIM; an ADASYN synthetic minority oversampling; and a logistic regression classifier with an L2 penalty. Increased performance was also evident on any calculated performance metric, except from the sensitivity (Table 2 and Figure 6).

Additionally, only subtle changes were presented between the exploratory and validation performances, as illustrated by a notable overlap on their polygon areas drawn using their corresponding radar plots (Figure 6).

**Figure 7:**
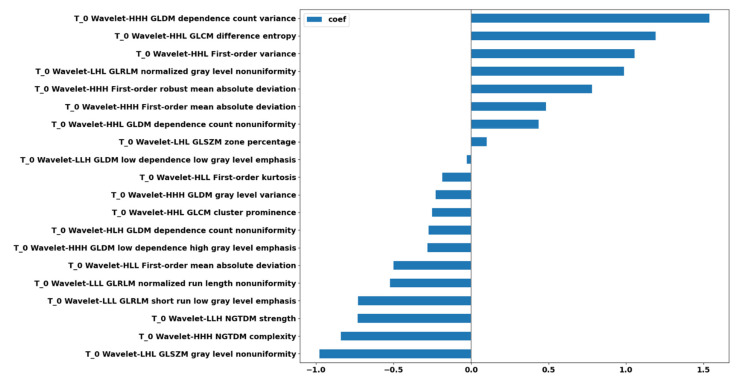
Feature ranking of the 20 selected radiomics features after feature reduction based on the regression coefficient profiles.

Of the 2499 p_MRI-based radiomics features, 20 features were found to be significant; all of them contributed to the prediction performance according to the L2 regularization of the logistic regression (Figure 7).

**Figure 8:**
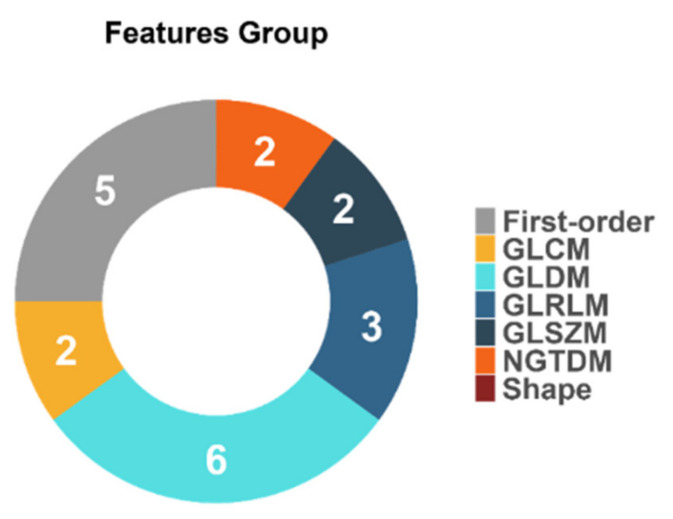
Feature distributions according to the group of the calculated radiomics features.

The descriptive statistics of the 20 selected features (Figure 8) depict the contribution of solely wavelet-based radiomics features extracted from images at time point T_0.

**Figure 9:**
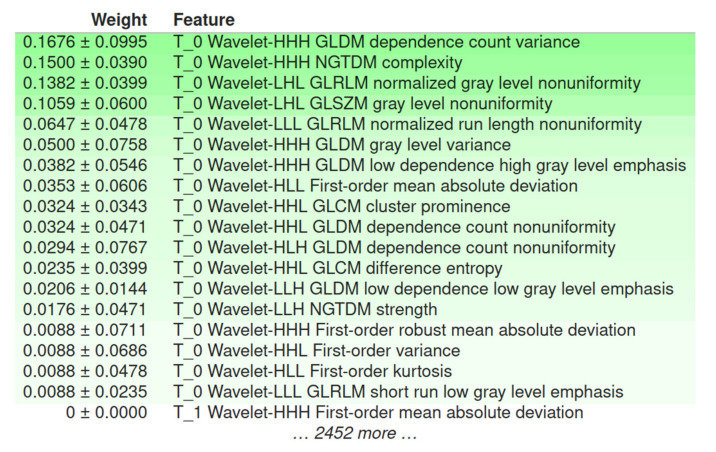
A top-down radiomics feature order in decreasing importance using local interpretable model-agnostic explanations (LIME). All radiomics features were shuffled individually and the permutation importance (reported as mean ± SD) was computed on the external validation set.

**Figure 10:**
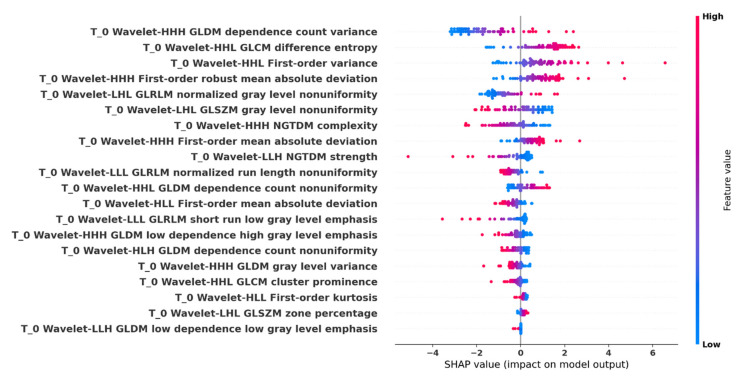
Shapley additive explanations (SHAP) summary plot. All important radiomics feature values are displayed as dots using a pseudocoloring (blue to red), low or zero contributors are near a SHAP value of zero, a long distance from zero denotes a higher influence of a specific feature in the prediction performance, where decreased or increased values favored the negative (IDH-mutant) or positive (IDH-wildtype) classes, respectively.

Global model interpretability of the optimal p_MRI-based radiomics analysis pipeline with regard to the prediction performance was obtained using LIME (Figure 9). Summary plot of the corresponding SHAP values is shown in Figure 10.

Assessment of the methodological quality of the presented radiomics study was conducted using the radiomics quality score (RQS), reaching a total of 14 out of 36 points (38.9%). Details are given in Table S2.

## 4. Discussion

This study investigated the prognostic value of DSC–MRI radiogenomics in gliomas regarding IDH mutation status prediction. Data from three different clinical centers (cohorts A, B, and C) were involved in the study; ML models were developed using cohort A and validated externally to demonstrate their generalizability on the independent cohorts B and C.

### 4.1. The Role of DSC–MRI Standardization

Radiogenomics IDH mutation status prediction is a promising but also challenging task that needs careful consideration when designing all the required steps of the analysis workflow. When initiating a multicenter radiomics study, the lack of a direct or modeled relationship between tissue and the MR signal intensities can yield a radiomics feature extraction from images that varies between different vendors and acquisition protocols. Published studies on phantoms have reported intensity-related bias that was measured to approach 100% in quantitative (pseudo-perfusion data) phantom studies due to the inability to correct for all image acquisition factors using postprocessing tools [33]. To address this issue, a dynamic-based MRI standardization was performed on perfusion data (p_MRI) obtained from T_0, T_max, and T_2 prior to radiomics and compared to the commonly used image postprocessing techniques (np_MRI). The performance results, outlined in the following text, showed that the radiomics from the proposed standardization method outperformed that of commonly-used techniques.

### 4.2. Performance of IDH Status Prediction and Informative Radiomics Features

A total of 2499 np_MRI and p_MRI based radiomics features, in compliance with IBSI guidelines, were calculated and subjected to the proposed analysis pipeline, revealing increased performance when using the proposed image standardization technique. Specifically, by applying a logistic regression with an L2 norm to the selected p_MRI-based radiomics features of the validation set, apart from the sensitivity in which a decrease of 18.14% was evident (np_MRI: 0.733, p_MRI: 0.600), we achieved a percentage change increase of 94.48% in the Cohen’s kappa metric (np_MRI: 0.145, p_MRI: 0.282), 14.22% in the F1-score (np_MRI: 0.415, p_MRI: 0.474), 4.38% in the AUC (np_MRI: 0.639, p_MRI: 0.667), 29.78% in the accuracy (np_MRI: 0.544, p_MRI: 0.706), and 49.90% in the specificity (np_MRI: 0.491, p_MRI: 0.736). Especially in the case of specificity, the np_MRI-based radiomics performance in the validation phase was lower than the baseline of 50%.

Feature selection on p_MRI data revealed a vital role of time point T_0 when making IDH status predictions since all 20 significant prognostic factors were calculated from images at that time point of the perfusion curve. This observation is in accordance with recent studies highlighting the importance of the initial components of the signal time–intensity curve for IDH status determination [34]. Signal deviation in these regions correlates to predictable vascularity phenotypes of IDH-mutant and IDH-wildtype tumors [35], as determined by IDH-specific vascular gene signatures [36]. Specifically, Choi et al. [34] demonstrated that the curve components between T_0 and T_max performed best for the identification of IDH-mutant versus IDH-wildtype gliomas by applying an explainable recurrent neural network to attribute attention weights to specific parts of the T2* signal time–intensity curve. IDH-wildtype tumors were characterized by a steeper downslope and larger signal drop in contrast to their IDH-mutant counterparts, which was compatible with the increased tumor angiogenesis in this group [22]. In the same study, the upslope of the signal drop (between T_max and T_2) and post-bolus plateau (beyond T_2) revealed a less steep and attenuated signal recovery in IDH-wildtype tumors, which likely reflected the increased permeability due to immature and leaky tumor vessels [22,34]. The latter observation was less apparent in our analysis, potentially due to the higher percentage of IDH-wildtype tumors in our patient population, which commonly confounds relevant studies and is in accordance with tumor epidemiology [1]. Such minuscule time-dependent differentiating traits between the two tumor groups regarding MR perfusion are potentially enhanced by MR data decomposition based on wavelet transform.

Another important observation was that all 20 significant predictors were calculated from decomposed MRI data after the wavelet transform. The inclusion of multi-resolution radiomics introduces band-pass frequency filtering encompassing smooth and detailed parts of the images. This result indicates that wavelet image decomposition enhanced the discriminatory power of radiomics, possibly due to the noise suppression and identification of frequency bands that better capture the actual imaging phenotype variability within the heterogeneous dataset. Our results confirmed the scale-dependent nature of texture and highlight its importance in multicenter studies, even if image standardization is included. Of the 20 selected wavelet-based features, 5 were from the first-order group and 15 were texture features (2 GLCM, 6 GLDM, 3 GLRLM, 2 GLSZM, and 2 NGTDM). Apparently, no shape descriptors were found to be important in the prediction. These results are in accordance with recent literature, highlighting the strong impact of the texture-based features in glioma IDH mutation status prediction [12]. According to the L2 logistic regression coefficient profiles, the feature ranking of these 20 radiomics features during the model development identified a significant predictive role of texture features that are sensitive to gray level variability, texture complexity, and non-uniformity. The most highly ranked radiomics features were found to be “dependence count variance” from GLDM, “difference entropy” from GLCM, and first-order “variance”.

### 4.3. Radiomics-Based ML Models’ Explainability

In light of the above observation, we applied model explainability mechanisms on the external validation phase using LIME and SHAP to evaluate and interpret the influence of the selected radiomics features in the radiomics analysis result. LIME verified the aforementioned results with a slight change in the features’ importance ranking, again with “dependence count variance” as the radiomics feature that most affected the predictive performance. Strongly associated with LIME, SHAP summary plot results indicated, among others, “dependence count variance” from GLDM, “difference entropy” from GLCM, and first-order “variance” as the strongest indicators of IDH status mutation prediction. The texture feature “dependence count variance” is sensitive to variability in voxel group sizes of similar intensity, implying increased complexity of the texture as its value increases, “difference entropy” is a texture disorder parameter that is analogous to the increase in the VOI’s signal intensity distribution differences, and “variance” reflects how gray levels are spread out about the mean. The first-order radiomic feature “robust mean absolute deviation” was also an important factor; it is associated with an increased contrast of high and low gray values between the 10th and 90th percentiles of the glioma histogram when the value increases. These characteristics of the dominant predictive radiomics can be explained using the more heterogeneous profile/nature of the IDH-wildtype glioma cases [22,34]. An analogous pattern was illustrated in the SHAP summary plot, where increased values of the top four features favored the IDH-wildtype mutation status (positive class) against the IDH-mutant status (negative class).

### 4.4. Limitations and Future Work

Several limitations were identified in the study, including the lack of radiomics features robustness assessment using a test–retest study. This is an important part of the radiomics quality score (RQS) questionnaire; however, this was not feasible due to the dynamic nature of the acquired data and the retrospective collection from all centers. Another important issue is the limited number of time points that were used to select data from the perfusion curve. Extending our current study, future work will focus on investigating the added value that might arise when changes in radiomics features between specific time points (i.e., similar to delta-radiomics) will be included in the analysis pipeline. Additionally, the next step will be to involve both radiomics features extracted from raw DSC–MRI data and the derived parametric maps (e.g., cerebral blood flow). A comparative study between the proposed dynamic-based standardization technique and efficient feature-based methods (i.e., radiomics features harmonization using ComBat [37]) will be an important task in our future work. Despite the promising results, the high imbalance favoring the IDH status mutation in the examined cohorts, and hence a small number of IDH-wildtype patients, both for the developing and validating the proposed radiomics analysis pipeline, was a major limitation of this study, calling for extended studies with more participating centers.

## 5. Conclusions

In conclusion, the proposed study highlighted the impact of DSC–MRI radiogenomics in gliomas for IDH status subtyping. Increased accuracy was observed when dynamic-based standardization of the acquired imaging data was performed prior to radiomics and the generalizability of the proposed radiomics analysis was confirmed using two independent validation sets. Model explainability mechanisms discovered intuitive patterns between the IDH mutation status and radiomics features, which enhances the potential clinical value of the proposed DSC–MRI-based radiogenomics.

## Figures and Tables

**Table 1:** Imaging protocol parameters for the patient population.

	Cohort A	Cohort B		Cohort C
Scanner Type	3T Discovery MR750 (GE Healthcare, Chicago, Illinois, United States)	3T Siemens Skyra (Siemens Healthineers, Erlangen, Germany)	1.5T Magnetom Avanto (Siemens Healthineers, Erlangen, Germany)	1.5.T Philips Achieva (Philips Medical Systems, Eindhoven, Netherlands)
Acquisition type	2D Echo-Planar Imaging (EPI) with fat suppression (FS)			
Magnetic field strength	3T	3T	1.5T	1.5T
Repetition time (ms)	1800	1870	1850	1525
Echo time (ms)	40	30	30	40
Echo train length	1	63	1	47
Flip angle (deg)	60	90	90	75
In-plane resolution (mm^2^)	1.718 × 1.718	1.719 × 1.719	1.796 × 1.796	1.75 × 1.75
Number of averages	1	1	1	1
Image slice thickness (mm)	5	5	5	5
Image slice spacing (mm)	5	5	5	5
Temporal resolution	60 × 1.87 s	60 × 2.07 s	40 × 1.53 s	60 × 1.8 s
Matrix size	128 × 128	128 × 128	128 × 128	128 × 128

**Table 2:** Performance of np_MRI and p_MRI radiomics in isocitrate dehydrogenase (IDH) mutation status prediction.

Performance Metrics	Exploratory Set		Validation Set	
	np_MRI	p_MRI	np_MRI	p_MRI
Cohen’s kappa	0.193 ± 0.096	0.303 ± 0.125	0.145	0.282
F1-score	0.482 ± 0.163	0.533 ± 0.164	0.415	0.474
AUC	0.618 ± 0.148	0.678 ± 0.103	0.639	0.667
Accuracy	0.587 ± 0.144	0.707 ± 0.108	0.544	0.706
Sensitivity	0.689 ± 0.208	0.611 ± 0.183	0.733	0.600
Specificity	0.547 ± 0.189	0.745 ± 0.146	0.491	0.736
PPV	0.388 ± 0.150	0.508 ± 0.196	0.290	0.391
NPV	0.823 ± 0.158	0.834 ± 0.095	0.868	0.868

AUC, area under the curve; PPV, positive predictive value; NPV, negative predictive value.

## Data Availability

The datasets used and analyzed during the current study, and the code for the machine learning and the radiomics analysis are available from the corresponding author on reasonable request.

## Supplementary Materials

The following are available online at https://www.mdpi.com/article/10.3390/cancers13163965/s1, Table S1: List of the calculated features using Pyradiomics, Table S2: Methodological quality assessment of the proposed study using the radiomics quality score (RQS).
