# Supplementary material for: Multicenter DSC–MRI-Based Radiomics Predict IDH Mutation in Gliomas
Source: Cancers (Basel). 2021 Aug 5;13(16):3965. doi: 10.3390/cancers13163965 (PMC8391559; doi:10.3390/cancers13163965)
Supplement: Supplementary file 1 [file cancers-13-03965-s001.zip › cancers-1292538-supplementary/Supplemental Table 1.pdf]

Table S1. List of the calculated features using Pyradiomics.

Group	Short Description	Features Name
Group 1	First-order statistics	Mean<br>Variance<br>Skewness<br>Kurtosis<br>Median<br>10th percentile<br>90th percentile<br>Interquartile range<br>Range<br>Mean absolute deviation<br>Robust mean absolute deviation<br>Entropy<br>Energy<br>Root mean square<br>Total energy<br>Uniformity
Group 2	Shape descriptors	Volume (voxel counting)<br>Volume (mesh)<br>Surface area (mesh)<br>Surface to volume ratio<br>Elongation<br>Flatness<br>Least axis length<br>Major axis length<br>Minor axis length<br>Maximum 3D diameter<br>Maximum 2D diameter (Column)<br>Maximum 2D diameter (Row)<br>Maximum 2D diameter (Slice)<br>Sphericity
Group 3	Co-occurrence matrix features (GLCM: Gray Level Co-occurrence Matrix)	Autocorrelation<br>Cluster prominence<br>Cluster shade<br>Cluster tendency<br>Contrast<br>Correlation<br>Difference average<br>Difference entropy<br>Difference variance<br>Inverse difference<br>Inverse difference moment<br>Normalized inverse difference moment<br>Normalized inverse difference<br>Inverse variance<br>Information correlation 1<br>Information correlation 2<br>Joint average<br>Joint energy

		Joint entropy<br>Maximal correlation coefficient<br>Maximum probability<br>Sum average<br>Sum entropy<br>Sum squares
Group 4	Neighboring gray level dependence matrix features (GLDM: Gray Level Dependence Matrix)	Dependence count entropy<br>Dependence count variance<br>Dependence count nonuniformity<br>Normalized dependence count nonuniformity<br>Gray level nonuniformity<br>Gray level variance<br>High gray level count emphasis<br>Low gray level count emphasis<br>Low dependence emphasis<br>High dependence emphasis<br>High dependence high gray level emphasis<br>High dependence low gray level emphasis<br>Low dependence low gray level emphasis<br>Low dependence high gray level emphasis
	Size zone matrix features (GLSZM: Gray Level Size Zone Matrix)	Gray level nonuniformity<br>Normalized gray level nonuniformity<br>Gray level variance<br>Large zone emphasis<br>Small zone emphasis<br>Zone percentage<br>Zone size entropy<br>Zone size variance<br>Zone size nonuniformity<br>Normalized zone size nonuniformity<br>Low gray level emphasis<br>High gray level emphasis<br>Large zone high gray level emphasis<br>Large zone low gray level emphasis<br>Small zone high gray level emphasis<br>Small zone low gray level emphasis
	Run length matrix features (GLRLM: Gray Level Run Length Matrix)	Short runs emphasis<br>Long runs emphasis<br>Low gray level run emphasis<br>High gray level run emphasis<br>Short run low gray level emphasis<br>Short run high gray level emphasis<br>Long run low gray level emphasis<br>Long run high gray level emphasis<br>Gray level nonuniformity<br>Normalized gray level nonuniformity<br>Gray level variance<br>Run entropy<br>Run percentage<br>Run length variance<br>Run length nonuniformity

	Normalized run length nonuniformity
Neighborhood gray tone difference matrix features (NGTDM: Neighbouring Gray Tone Difference Matrix)	Coarseness<br>Contrast<br>Busyness<br>Complexity<br>Strength
