# Supplementary material for: Multicenter DSC–MRI-Based Radiomics Predict IDH Mutation in Gliomas
Source: Cancers (Basel). 2021 Aug 5;13(16):3965. doi: 10.3390/cancers13163965 (PMC8391559; doi:10.3390/cancers13163965)
Supplement: Supplementary file 1 [file cancers-13-03965-s001.zip › cancers-1292538-supplementary/Supplemental Table 2.pdf]

Table S2. Methodological quality assessment of the proposed study using the radiomics quality score (RQS).

RQS checkpoint	Criteria	Points
First	Image protocol quality	+1
Second	Multiple segmentations	+1
	Phantom study	+0
	Multiple time points	+0
Third	Feature reduction or adjustment for multiple testing	+3
	Multivariate analysis with non radiomics features	+0
	Biological correlates	+1
	Cut-off analysis	+0
	Discrimination statistics	+2
	Calibration statistics	+0
	Prospective study registered in a trial database	+0
	Validation	+4
	Comparison to "gold standard"	+0
	Potential clinical application	+2
	Cost-effectiveness analysis	+0
	Open science and data	+0
**Total points achieved**		14/36
